# Mortality of Various Cities

**Published:** 1874-04

**Authors:** 


### MORTALITY OF VARIOUS CITIES.

The *Popular Science Monthly* says: Dr. Charles P. Russel] gives a tabulated statement of the mortality of the various States of the union, from which we borrow the following regarding the death rates of various cities: The highest death rate in 1873 was exhibited by Memphis, where the deaths were 46.6 in each 1000 inhabitants. Other cities followed in this order: Savanah, 39.2; Vicksburg, 36.5; Troy, 34; Hoboken. 39.9; New York, 32.7; Newark, 31.6; New Orleans, 30.6; Boston, 30.5. The rate for Philadelphia was only 26.1; Brooklyn, 28.1; St. Louis, 21; Chicago, 27.6; Baltimore, 25.1; Cincinnati, 20.5; San Francisco, 17.2. This compares not unfavorably with the mortuary statistics of British cities, where the lowest rate was 21.4; that of London, Bombay and Calcutta, only 29.2 and 25, respectively. The highest known death rate prevailed in Valparaiso, Chili, 66.9.

